# Life Skills Link to Mind Wandering Among University Students: An Exploratory Study

**DOI:** 10.3389/fpsyg.2021.729898

**Published:** 2021-10-11

**Authors:** Takayoshi Kase, Toshikazu Kawagoe

**Affiliations:** ^1^College of Contemporary Psychology, Rikkyo University, Saitama, Japan; ^2^Liberal Arts Education Center, Kyushu Campuses, Tokai University, Kumamoto, Japan

**Keywords:** mind wandering, life skills, exploratory, qualitative, text mining

## Abstract

The occurrence of mind wandering (MW) leads to lower performance on memory tasks related to lecture contents in educational settings, which has been recognized as problematic. To date, several dispositional factors have been reported as being associated with MW. This study investigated whether another psychological component—life skills—is linked to MW. Specifically, it clarified the relationship between life skills and two types of MW: state MW (occurs while performing a given task) and trait MW (occurs subjectively in daily life), using a sample of university students. From the perspective of cognitive and emotional control functions, life skills are thought to be related to the occurrence of MW. In addition to common questionnaire surveys, by recording and analyzing the participants' self-reports for MW occurrence during the experimental task, we clarified not only the quantitative associations among the variables but also the qualitative differences. Multiple regression analysis for the data from 53 students showed that decision-making and coping-with-emotion skills are negatively related to the occurrence of mind wandering. The qualitative data additionally revealed that participants with high decision-making skills are more likely than those with low decision-making skills to attempt to maintain their concentration on the task by thinking about task execution. These results suggest that life skills are associated with MW and that the ability to inhibit MW may be enhanced by improving life skills because they comprise acquired, learnable behaviors and attitudes. Life skills training may help in reducing students' MW in educational contexts.

## Introduction

Mind wandering (MW) refers to a phenomenon in which attention is diverted from the task at hand and transferred to internal processes, such as thoughts and sensations, which may be unrelated to the task (Smallwood and Schooler, 2006, 2015). MW has been reported to be functional, including creative thinking and problem solving (Baird et al., 2012). However, it has also been found to have negative impacts, such as decreasing concentration on tasks, and has been associated with negative consequences, including medical errors (Berner, 2011; for more information about the pros and cons of MW, see Mooneyham and Schooler, 2013). MW while performing tasks has been shown to reduce reading comprehension (Smallwood, 2011; Farley et al., 2013). In studies on university students, it has been reported that the higher the frequency of MW during a lecture, the lower the performance of memory tasks related to the lecture content (Lindquist and McLean, 2011; Risko et al., 2012). The occurrence of MW in educational settings has been recognized as problematic, and trials have been conducted to examine the method for identifying and controlling the factors leading to its occurrence in such contexts (Farley et al., 2013; Szpunar et al., 2013; Hattori and Ikeda, 2016).

When considering solutions to problems related to the occurrence of MW during task performance, other than improving the lecture materials and educational environment in which students work on their tasks, identifying the psychological factors related to the occurrence of MW could prove useful. One possible factor is life skills (LSs). LSs are defined as the abilities necessary to cope constructively and effectively with various problems and demands that arise in daily life (World Health Organization, 1994). It is a comprehensive concept that includes diverse cognitive skills necessary for individuals to demonstrate their capabilities in society, maintain mental health, and achieve a more fulfilling social life. LSs are acquired abilities and skills; they differ from dispositional factors such as personality traits (World Health Organization, 1994). The conceptual framework of LSs comprises psychological factors such as acquired learnable behaviors and attitudes that are subject to modeling in the social learning theory (Bandura, 1986; Botvin and Griffin, 2014). Therefore, LSs can be improved through psychoeducational programs called LSs training/education developed based on the social learning theory and problem behavior theory (World Health Organization, 1994; Botvin and Griffin, 2014; Prajapati et al., 2017). Based on the theoretical classification of competences that constitute LSs (Brooks, 1984; World Health Organization, 1994), Kase et al. (2016) identified the following four types that play a core role in the daily lives of adolescents and adults. First is decision-making skill (DM), which are abilities necessary for making judgments to solve problems effectively based on logic and imagination. Second is interpersonal relationship skill (IR), which are abilities required for imagining feelings and emotions from others' words and actions and for expressing empathy. Third is effective communication skill (EC), which are competences necessary for actively and effectively communicating one's thoughts to others. Fourth is coping-with-emotions skill (CE), which entail abilities for managing one's own emotions efficiently (Kase et al., 2016). Of these, DM skills include functions of cognitive control, such as planning and organizing information, and CE skills involve switching to a positive mood (Kase et al., 2016).

People with higher LSs are more likely to engage in socially adaptive behavior (World Health Organization, 1994; Botvin and Griffin, 2014). In educational settings, those with higher LSs may also exhibit adaptive behaviors such as less MW during a lecture. Conceptually, DM can be considered as reflecting executive functions and CE as affecting emotional control; as such, these factors appear to be associated with the occurrence of MW (Smallwood et al., 2009; McVay and Kane, 2010; Hattori and Ikeda, 2016). Meanwhile, theoretically speaking, IR and EC may not affect MW. Although previous findings have shown that dispositional factors such as current concern (McVay and Kane, 2010), conscientiousness (Nicosia and Balota, 2021), and motivation (Seli et al., 2019; Kawagoe et al., 2020) affect MW, it is unclear whether LSs have any relevance to MW. Their relationship is probable because there are common typical indices for MW and LSs such as concentration on the given tasks and lectures (Brooks, 1984; Berner, 2011; Smallwood and Schooler, 2015). Mood is another important factor that affects MW (Mrazek et al., 2013; Smallwood and Schooler, 2015; Hattori and Ikeda, 2016), and its associations with MW are similar to that of motivation, wherein the higher the motivation, the higher the rate of MW or LSs (Hardcastle et al., 2015; Seli et al., 2019; Kawagoe et al., 2020). In this study, we investigate the association between LSs and the tendency of university students to engage in MW.

This study speculated that LSs, including DM and CE skills, are related to the tendency of university students to engage in MW while performing a given task and in their daily life. To explore this possibility, in addition to the self-report of the students' MW experience in daily life, we used a method that allowed us to examine empirically the contents of MW in detail. This method is open-ended and can be considered as the most unbiased way of collecting and probing MW data (Kawagoe and Kase, 2020). By recording and analyzing the reports of participants' self-reflection during the experiment, we clarified not only the quantitative associations among the variables but also the qualitative differences, to strengthen the study's findings and their implications.

## Materials and Methods

### Participants

The participants of this study were almost the same as in Kawagoe and Kase (2020). Fifty-five participants were recruited using opportunity sampling during a semester at Rikkyo University. Two of them were undergoing psychiatric treatment, and one was unable to complete the entire experiment. Finally, the 53 participants [47 women and 6 men; mean age, 19.2 years (age range: 18–23)] were included in this study; all of them provided written informed consent before participating in the study. Because of the sampling method, the study had a small sample size, which reduced the power of the study and increased the margin of error. However, the current sample size met the minimum criteria for regression analysis (Jenkins and Quintana-Ascencio, 2020). The study was conducted in accordance with the World Medical Association (2013), and the study protocol was approved by the ethics committee of the Department of Contemporary Psychology, Rikkyo University.

### Experimental Task

We used a single experimental task to assess the occurrence of MW and its content. A sustained attention response task (SART) using the experience sampling method (Robertson et al., 1997) was employed. In this task, the participants were instructed not to press a button if they observe a target with low frequency (i.e., “4”) embedded in a series of stimuli that they were supposed to respond to (i.e., respond to the numbers from “1” to “9,” except the target “4”). During this task, they were randomly and abruptly asked, “How much did your mind wander just before this probe?” The participants were required to respond to this question on a 7-point Likert scale, where 1 = “not at all” and 7 = “strongly.” During the experiment, they were asked this question 15 times, and after a button was pressed, the participants were instructed to elaborate on what they had been thinking about immediately before the probe was presented. The experimenter dictated the verbal report in a way that was as accurate and unbiased as possible, and responded to queries as needed. The protocol of this task was identical to our previous study; as such, further details and the rationale for this open-ended method to assess MW can be found in Kawagoe and Kase (2020).

### Measures

In this study, we differentiated between “state” and “trait” MW. Since these two concepts are weakly intercorrelated (Seli et al., 2016; Kawagoe et al., 2020), their associations with external variables would differ. State MW refers to in-the-moment reactions to internal or external stimuli or situations, whereas trait MW is part of an individual's personality and therefore, is a long-term characteristic of the individual. As a quantitative index of the level of state MW during the task, the mean value of the scores of the 15 probes during the SART was calculated (Probe MW). In addition, we obtained the MW reports for the participants' experiences during SART via an open-ended method. We focused on the subjective reports but not the behavioral results on the SART to minimize the risk for type I error by restricting the number of statistical tests. These reports were analyzed as qualitative data, and are presented in detail in Table S1 in Kawagoe and Kase (2020). Meanwhile, the participants' trait MW was measured using the Mind Wandering Questionnaire (MWQ; Mrazek et al., 2013). It comprises five items (e.g., “I have difficulty maintaining focus on simple or repetitive work”), rated on a 7-point Likert scale, with higher total scores indicating higher levels of MW.

LSs were assessed using the Life Skills Scale for Adolescents and Adults (LSSAA; Kase et al., 2016). It consists of 21 items across four subscales: DM (eight items; e.g., “I can think carefully about the pros and cons of things”), IR (five items; e.g., “When listening to others, I am able to imagine their feelings by putting myself in their position”), EC (five items; e.g., “I can clearly convey my true feelings to others”), and CE (three items; e.g., “I am able to control my emotions, for example, by calming myself down”). Each item is assessed on a 5-point Likert scale, with higher total scores indicating greater levels of LSs. The LSSAA items were developed based on free-description type questionnaires and text mining from adolescents and adults living in Japan (Kase et al., 2016), as well as the framework of LSs proposed by the World Health Organization (1994).

### Statistical Analysis

After the descriptive statistics and reliability coefficients (McDonald's ω) for each variable were calculated, a multiple regression analysis by the forced entry method was performed, with each subscale score of the LSSAA as the independent variables and the scores for the MWQ and Probe MW as the dependent variables. These analyses were conducted using the Statistical Package for Social Sciences (version 26; IBM, Armonk, NY, USA).

For qualitative analysis, the texts of the oral reports collected during the SART were analyzed using a co-occurrence network analysis (Osgood, 1959; Danowski, 1993; Higuchi, 2014). It illustrates the relationships among the words in text data based on the Jaccard coefficient (Romesburg, 1984), which is an index indicating co-occurrence frequency. Co-occurrence network analysis using the Jaccard coefficient is used, for example, to organize the content of texts used in various media and to interpret overall trends (e.g., Chen et al., 2021). The Jaccard coefficient value ranges from 0 to 1, and the closer the value is to the latter, the stronger the association between the two sets of words, *A* and *B*. It is calculated using the following equation:


$$\begin{array}{l} J\left(A,B\right)=\frac{A\cap B}{A\cup B} \end{array}$$

To elucidate the qualitative relationship between the contents of MW and LSs, the participants were split into two: those who scored above the mean of each subscale (DM and CE) of the LSSAA were classified as the high group, and those who scored below the mean were classified as the low group; then, the word frequency appearing in each group was illustrated as one network with Jaccard coefficients for each subscale. The number of words represented on a co-occurrence network was set to 30 in a descending order of the Jaccard coefficients. Using this method enabled us to understand the characteristics of the contents of MW during the SART in each group (high or low LSs). Before conducting these evaluations, the text data were pre-processed for text analysis, including correction of typographical errors, unification of expressions, and exclusion of uninterpretable words. The co-occurrence network analysis was conducted using KH Coder, version 3 (Higuchi, 2014).

## Results

The descriptive statistics of each variable are shown in Table 1. There were no ceiling or floor effects in the descriptive statistics and reliability coefficients. The values of McDonald's ω of the EC subscale in the LSSAA and the MWQ were below 0.70, and those of the other scales were above this value. The values of the correlation coefficients between all variables ranged from |0.014| to |0.336|. As shown in Table 2, the multiple regression analysis indicates that the score for CE is negatively correlated with that for the MWQ (β = −0.278, 95% CI: −0.555, −0.001, *p* = 0.049), and the score for DM is negatively related to the score for Probe MW (β = −0.331, 95% CI: −0.618, −0.044, *p* = 0.025). The range of the variance inflation factor values was 1.080–1.186 (Table 2), indicating no multi-collinearity among all independent variables.

**Table 1:** Descriptive statistics for each study variable (*n* = 53).

		**Mean**	***SD***	**95% CI**		**McDonald's** **ω**
				**Lower**	**Upper**	
LSSAA	DM	3.46	0.68	3.27	3.65	0.82
	IR	3.85	0.66	3.66	4.03	0.72
	EC	3.09	0.66	2.91	3.28	0.62
	CE	2.87	0.92	2.62	3.13	0.76
MWQ		3.82	0.70	3.63	4.01	0.58
Probe MW		1.56	0.61	1.40	1.73	–

*CI, confidence interval; DM, decision-making skills; IR, interpersonal relationship skills; EC, effective communication skills; CE, coping with emotion skills; MWQ, Mind Wandering Questionnaire; Probe MW, probe mind wandering; LSSAA, Life Skills Scale for Adolescents and Adults*.

**Table 2:** Multiple linear regression for the index of mind wandering (*n* = 53).

	**MWQ (trait mind wandering)**					**Probe mind wandering (state mind wandering)**					**VIF**
	**β**	**95% CI**		***t***	***P***	**β**	**95% CI**		***t***	***p***	
		**Lower**	**Upper**				**Lower**	**Upper**			
Age	0.077	−0.207	0.361	0.545	0.588	−0.228	−0.512	0.057	−1.608	0.115	1.169
Sex	0.139	−0.134	0.412	1.027	0.310	0.022	−0.252	0.296	0.164	0.870	1.082
DM	−0.266	−0.552	0.020	−1.870	0.068	−0.311	−0.618	−0.044	−2.320	0.025	1.186
IR	−0.113	−0.390	0.164	−0.820	0.416	0.222	−0.056	0.500	1.609	0.115	1.112
EC	0.032	−0.240	0.305	0.239	0.812	0.073	−0.201	0.347	0.539	0.593	1.080
CE	−0.278	−0.555	−0.001	−2.023	0.049	−0.047	−0.325	0.231	−0.341	0.735	1.111
*R* ^2^	0.218 (*p* = 0.067)					0.212 (*p* = 0.076)					

*β means standardized beta; MWQ, Mind Wondering Questionnaire; CI, confidence interval; DM, decision-making skills; IR, interpersonal relationship skills; EC, effective communication skills; CE, coping with emotion skills; VIF, variance inflation factor*.

**Figure 1:**
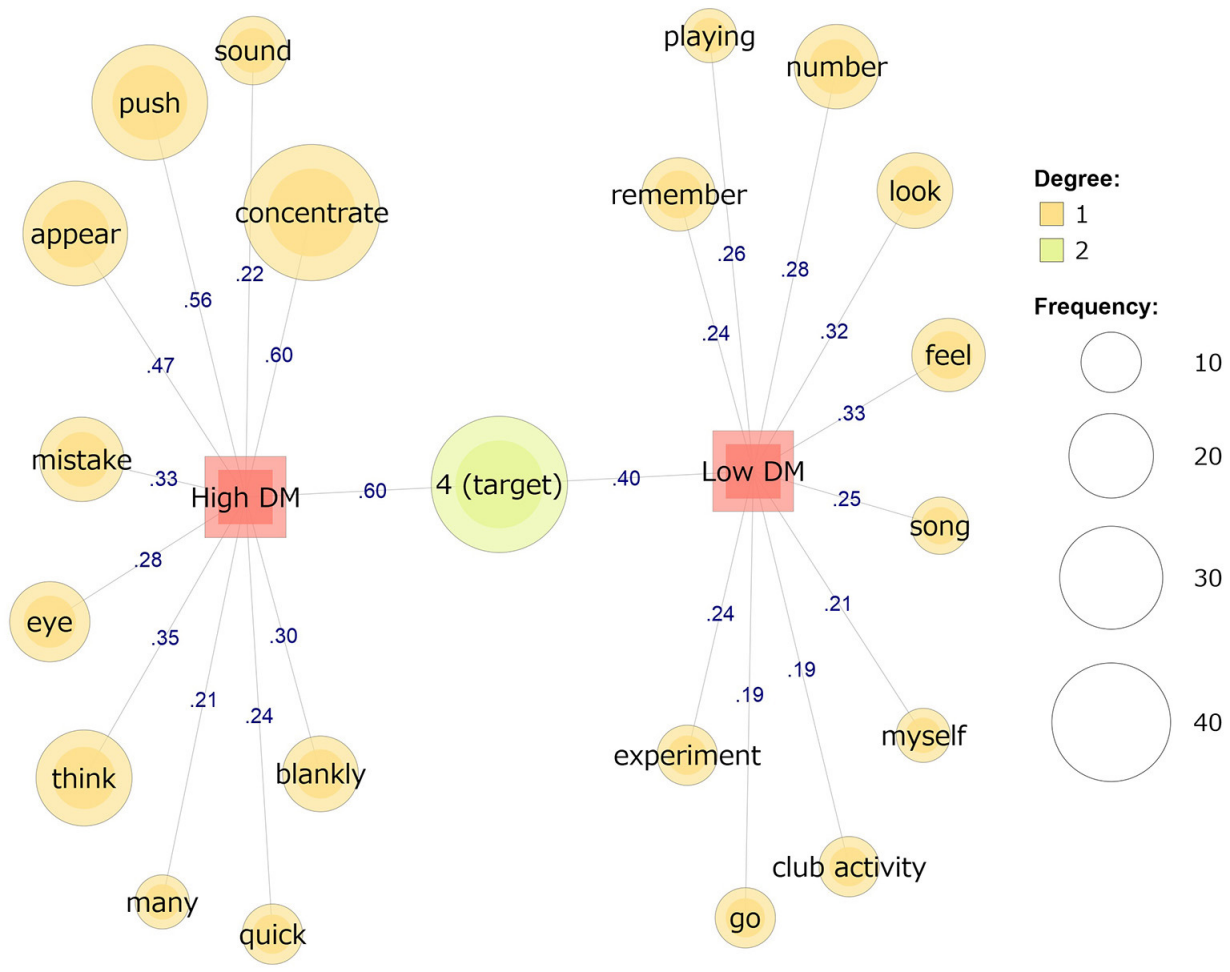
Comparison of the co-occurrence networks for self-reported mind wandering between the high (*n* = 32) and low (*n* = 21) groups for decision-making skills. The values on the line are Jaccard coefficients.

Co-occurrence network analysis for the reports during SART was performed for the DM subscale, which showed a significant relationship with the Probe MW scores. There was a significant difference between the high and low groups for both DM [high group: mean = 3.934, *SD* = 0.374; low group: mean = 2.738, *SD* = 0.301; *t*(48.79) = 12.827, *p* < 0.001) and CE (high group: mean = 3.586, *SD* = 0.501; low group: mean = 2.014, *SD* = 0.456; *t*(50.52) = 11.951, *p* < 0.001). The results of the co-occurrence network analysis are shown in Figure 1 and Table 3. First, in the high DM group (*n* = 32), “concentration,” “appear,” and “mistake” were found to be the typical words (Jaccard coefficients ≥ 0.33), all of which seemed to be associated with the given task (i.e., SART). In the low DM group (*n* = 21), “song,” “flowing,” “remember,” “myself,” and “club activity” appeared as the typical words (Jaccard coefficients ≥ 0.19), which seemed to be associated with occurrences of MW (Figure 1). Only “target” was a common word in both groups.

**Table 3:** Example of the reports for each word in the co-occurrence network analysis for decision-making skills.

**Group**	**Word**	**Example report**
High DM	Sound	I was distracted by the sound of the air conditioner.
	Push	I will be more accurate if I look at the number and push the button quickly.
	Appear	“4” appears more often.
	Concentrate	I need to concentrate on the task.
	Mistake	I pushed button “4” by mistake.
	Eye	I have to keep my eyes from drying out.
	Think	I might be able to avoid mistakes by thinking of the numbers as symbols and only considering the form “4.”
	Many	“4” appears many times.
	Blankly	I gazed blankly at the numbers.
	Quick	I will be more accurate if I look at the number and push the button quickly.
Low DM	Playing	My favorite song was playing in my head.
	Number	What is the mechanism of the appearance of these numbers?
	Remember	I made a mistake in “4” and remembered the time I made a mistake in my part-time job.
	Feel	I feel sleepy.
	Look	I looked at the screen too much and it was becoming difficult to see the vertical lines on the frame of the display.
	Song	My favorite song was flowing in my head.
	Myself	I was thinking about myself of myself at an old age.
	Club activity	I don't want to attend the club activities after this experiment because the practice is too hard.
	Go	I was thinking about the current weather. I want to go back before the rain.
	Experiment	I was thinking about what I will do after this experiment.

*DM, decision-making skills*.

**Figure 2:**
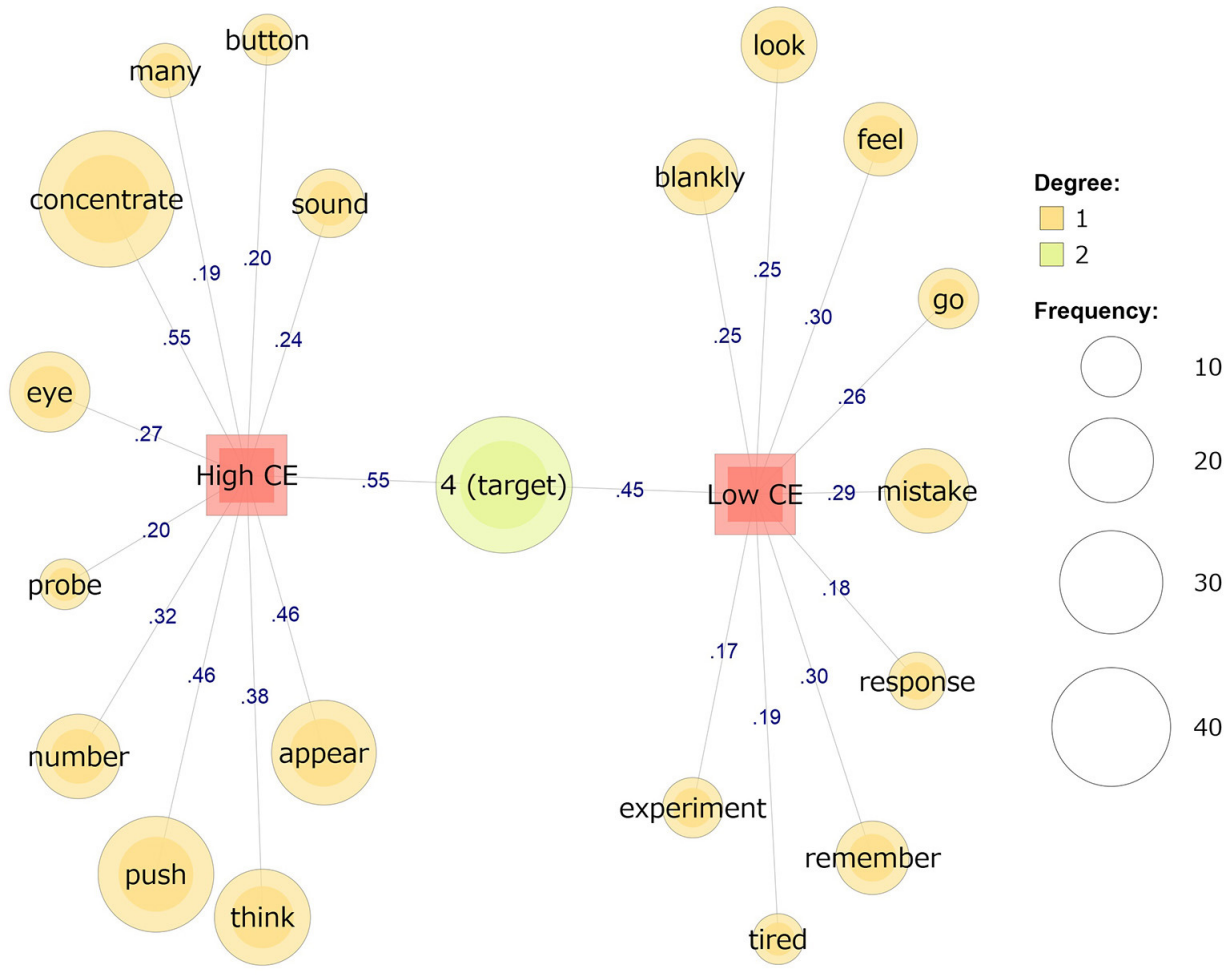
Comparison of the co-occurrence networks for self-reported mind wandering between the high (*n* = 29) and low (*n* = 24) groups for coping-with-emotion skills. The values on the line are Jaccard coefficients.

The same analysis was performed for CE, although the regression analysis was not significant for state MW. In the high CE group (*n* = 29), “push,” “appear,” “thinking,” “button,” and “probe” were the typical words (Jaccard coefficients ≥ 0.20). In the low CE group (*n* = 24), “think,” “remember,” and “tired” were found to be the typical words (Jaccard coefficients ≥ 0.19) (Figure 2; Table 4).

**Table 4:** Example of the reports for each word in the co-occurrence network analysis for coping-with-emotion skills.

**Group**	**Word**	**Example report**
High CE	Button	I feel good because I can restrict the button press to “4.”
	Many	“4” appears many times.
	Concentrate	I need to concentrate on the task.
	Sound	I was listening to the sound of the air conditioner.
	Eye	I have to keep my eyes from drying out.
	Probe	I was thinking what the hilarious answers to the probes will be?
	Number	I will be more accurate if I look at the number and push the button quickly.
	Push	I will be more accurate if I look at the number and push the button quickly.
	Appear	“4” appears more often.
	Think	I might be able to avoid mistakes by thinking of the numbers as symbols and only considering the form “4”.
Low CE	Look	I looked at the screen too much and it was becoming difficult to see the vertical lines on the frame of the display.
	Feel	I feel sleepy.
	Blankly	I looked at the screen blankly.
	Go	I was thinking about the current weather. I want to go back before the rain.
	Mistake	I was remembering my past mistakes.
	Response	My response is getting slower.
	Remember	I was remembering my past mistakes.
	Tired	I'm tired. When will the task end?
	Experiment	I was thinking about what I will do after this experiment.

*CE, coping-with-emotion skills*.

## Discussion

This study investigated whether life skills is linked to MW among university students. We speculated that DM and CE skills are negatively related to MW, and we investigated this relationship using quantitative and qualitative data. The findings marginally support our hypothesis. Focusing on the quantitative associations between LSs and WM, we found that DM were negatively associated with state WM, and CE were negatively associated with trait MW, respectively. It has been reported that DM skills have short- (e.g., organizing information during conversations) and long-term functions (e.g., promoting well-planned behavior; Brooks, 1984; World Health Organization, 1994; Kase et al., 2016). The current results support these findings, in that DM was found to be associated with MW at both the short- and long-term levels, although the relationship between DM and trait MW did not reach a significant level (*p* = 0.068). Moreover, CE skills involve the ability to adjust permanently one's emotions in daily life to a positive direction according to the individual's characteristics (Brooks, 1984; World Health Organization, 1994; Kase et al., 2016). Our results also support these findings, as the CE's association was confirmed only at the trait level of MW.

The results of the text mining provided some qualitative reinforcement for the validity of these interpretations. First, the group with high DM skills demonstrated the will to “concentrate” (e.g., “I need to concentrate on the task”), “push” (e.g., “I will be more accurate if I look at the number and push the button quickly”), “appear” (e.g., “‘4' appears more often”), “thinking” (e.g., “I might be able to avoid mistakes by thinking of the numbers as symbols and only considering the form ‘4”'), and “quick” (e.g., “I will be more accurate if I look at the number and push the button quickly”). As shown in these examples, the participants reported many words that might have been relevant to the given task (i.e., SART). However, the group with low DM scores used words related to task execution, such as “song” and “flowing” (e.g., “My favorite song was flowing in my head”), “remember” (e.g., “I made a mistake in ‘4' and remembered the time I made a mistake in my part-time job”), “myself” (e.g., “I was thinking about myself after my old age”) and “club activity” (e.g., “I don't want to attend the club activities after this experiment because the practice is too hard”). They also reported “look” and “number;” these words seem to be task-related, but they were also used in contexts related to MW (e.g., “I ‘looked' at the screen too much and it was becoming difficult to see the vertical lines on the frame of the display,” and “What is the mechanism of the appearance of these numbers?”). The group with low DM scores tended to report several words that expressed thoughts that were not directly associated with the task itself. Hence, the results suggest that individuals with high DM skills attempt to maintain their concentration on a task by thinking about things directly related to its execution, unlike those with low DM skills. Although in this study, no significant quantitative relationship was found between CE skills and state MW, qualitative relationships were exploratively examined. The results of the co-occurrence network analysis can be taken to indicate the contents of MW that occurred during the task. In Figure 2, other than “tired,” there are no words that directly indicate emotions. This suggests that there was not much difference in the emotions experienced between the high and low CE groups. These results support the finding that CE skills do not have a quantitative association with state MW.

The present results suggest that the ability to inhibit MW could be strengthened by the enhancement of LSs. Training/education that can improve these LSs has been proposed as intra- and extra-curricular activities for university students (Minagawa et al., 2009; Savoji and Ganji, 2013), which can be an effective countermeasure against the type of MW that interferes with concentration in lectures. For instance, DM skills can be enhanced by using psychological scales to understand one's own personality traits; following which, worksheets to set student life goals that match such traits can be used (World Health Organization, 1994; Eisen et al., 2003; Minagawa et al., 2009). Meanwhile, CE skills can be improved through group work, role-playing (in which participants talk about their emotions and listen to those of others), and through training, to increase self-esteem and respect for others (World Health Organization, 1994; Minagawa et al., 2009; Vatankhah et al., 2014). Implementing LSs training programs focused on both DM and CE skills can constitute a comprehensive attempt to reduce the occurrence of unintentional MW in everyday life, as well as during lectures and other task-performing situations.

The current study indicates that DM skills are negatively related to state MW and CE skills, to trait MW. By using qualitative analysis, we were able to clarify the relationship between MW and LSs based on the content of MW that occurred during the execution of the experimental task. Our results suggest the importance of LSs, which although relatively vague, are still a comprehensive concept for skills required for daily living in the context of MW. MW may be controlled via life skills training through the DM and CE components.

### Limitations and Future Directions

Since we explored the possible association between MW and LSs using an opportunity sampling method among university students, the sample size was not sufficient to obtain statistics with high estimation accuracy. Although the current sample size satisfies the minimum recommended sample size (Jenkins and Quintana-Ascencio, 2020), a larger sample size could improve the qualitative data and strengthen the differences observed in the participants' MW experience attributable to their LSs. This study was also limited to examining the relationship between MW that occurs during experimental tasks and LS. Future studies need to deal with MW that occurs during the performance of tasks directly related to lectures, such as reading materials and taking tests, to obtain findings that have more significance to problems related to MW among university students. Moreover, as noted in the Introduction section, several confounding factors likely existed such as current concern, conscientiousness, motivation, and mood. Task interests and age can also be other confounders. To conclude that LSs training can reduce MW, more sophisticated studies that focus on these factors are needed.

## Data Availability Statement

The raw data supporting the conclusions of this article will be made available by the authors, without undue reservation.

## Ethics Statement

The studies involving human participants were reviewed and approved by the Ethics Committee of the Department of Contemporary Psychology, Rikkyo University. The patients/participants provided their written informed consent to participate in this study.

## Author Contributions

TKas and TKaw conceived the idea presented in this study, developed the theory, and verified it using analytical methods. TKaw carried out the experiment and TKas conducted the analysis and prepared the first version of the manuscript. All authors discussed the results and contributed to the final manuscript.

## Funding

This work was supported by the JSPS KAKENHI (Grant Numbers: 19K14393 and 19K14481).

## Conflict of Interest

The authors declare that the research was conducted in the absence of any commercial or financial relationships that could be construed as a potential conflict of interest.

## Publisher's Note

All claims expressed in this article are solely those of the authors and do not necessarily represent those of their affiliated organizations, or those of the publisher, the editors and the reviewers. Any product that may be evaluated in this article, or claim that may be made by its manufacturer, is not guaranteed or endorsed by the publisher.
